# The influence of depressive symptoms and school-going status on risky behaviors: a pooled analysis among adolescents in six sub-Saharan African countries

**DOI:** 10.3389/fpsyt.2023.1171231

**Published:** 2023-07-24

**Authors:** Uttara Partap, Nega Assefa, Yemane Berhane, Ali Sie, David Guwatudde, Japhet Killewo, Ayoade Oduola, Mary M. Sando, Said Vuai, Richard Adanu, Till Bärnighausen, Wafaie W. Fawzi

**Affiliations:** ^1^Department of Global Health and Population, Harvard T.H. Chan School of Public Health, Boston, MA, United States; ^2^School of Public Health, College of Health and Medical Sciences, Haramaya University, Dire Dawa, Ethiopia; ^3^Addis Continental Institute of Public Health, Addis Ababa, Ethiopia; ^4^Nouna Health Research Center, Nouna, Burkina Faso; ^5^Department of Epidemiology and Biostatistics, Makerere University School of Public Health, Kampala, Uganda; ^6^Department of Epidemiology and Biostatistics, Muhimbili University of Health and Allied Sciences, Dar es Salaam, Tanzania; ^7^University of Ibadan Research Foundation, University of Ibadan, Ibadan, Nigeria; ^8^Africa Academy for Public Health, Dar es Salaam, Tanzania; ^9^College of Natural and Mathematical Sciences, University of Dodoma, Dodoma, Tanzania; ^10^Department of Population, Family and Reproductive Health, University of Ghana School of Public Health, Accra, Ghana; ^11^Heidelberg Institute of Global Health, University of Heidelberg, Heidelberg, Germany; ^12^Africa Health Research Institute, KwaZulu-Natal, South Africa; ^13^Department of Epidemiology, Harvard T.H. Chan School of Public Health, Boston, MA, United States; ^14^Department of Nutrition, Harvard T.H. Chan School of Public Health, Boston, MA, United States

**Keywords:** sub-Saharan Africa, adolescents, risky behavior, depressive symptoms, schools

## Abstract

**Background:**

Evidence from sub-Saharan Africa (SSA) regarding risky behaviors among adolescents remains scarce, despite the large population (approximately 249 million out of 1.2 billion globally in 2019) of adolescents in the region. We aimed to examine the potential influence of depressive symptoms and school-going status on risky behaviors among adolescents in six SSA countries.

**Methods:**

We used individual cross-sectional data from adolescents aged 10–19 based in eight communities across six SSA countries, participating in the ARISE Network Adolescent Health Study (*N* = 7,661). Outcomes of interest were cigarette or tobacco use, alcohol use, other substance use, getting into a physical fight, no condom use during last sexual intercourse, and suicidal behavior. We examined the proportion of adolescents reporting these behaviors, and examined potential effects of depressive symptoms [tertiles of 6-item Kutcher Adolescent Depression Scale (KADS-6) score] and school-going status on these behaviors using mixed-effects Poisson regression models. We also assessed effect modification of associations by sex, age, and school-going status.

**Results:**

The proportion of adolescents reporting risky behaviors was varied, from 2.2% for suicidal behaviors to 26.2% for getting into a physical fight. Being in the higher tertiles of KADS-6 score was associated with increased risk of almost all risky behaviors [adjusted risk ratio (RR) for highest KADS-6 tertile for alcohol use: 1.70, 95% confidence interval (95% CI): 1.48–1.95, *p* < 0.001; for physical fight: 1.52, 95% CI: 1.36–1.70, *p* < 0.001; for suicidal behavior: 7.07, 95% CI: 2.69–18.57, *p* < 0.001]. Being in school was associated with reduced risk of substance use (RR for alcohol use: 0.73, 95% CI: 0.53–1.00, *p* = 0.047), and not using a condom (RR: 0.81, 95% CI: 0.66–0.99, *p* = 0.040). There was evidence of modification of the effect of school-going status on risky behaviors by age and sex.

**Conclusion:**

Our findings reinforce the need for a greater focus on risky behaviors among adolescents in SSA. Addressing depressive symptoms among adolescents, facilitating school attendance and using schools as platforms to improve health may help reduce risky behaviors in this population. Further research is also required to better assess the potential bidirectionality of associations.

## Introduction

Adolescence has been identified as an important period during which engagement in risk-taking substantially increases (1–4). Risk-taking is understood to be a normal part of adolescent behavior, driven by biological processes including brain development, alongside other social and environmental influences (3, 5). Healthy risk taking comprises of generally low-risk, socially acceptable activities that contribute to improved adolescent development and well-being, such as trying a new sport (3, 4). On the other hand, unhealthy risk taking, also referred to as risky behaviors, include behaviors that expose individuals to increased likelihood of adverse consequences that substantially outweigh any potential benefit, and that may negatively affect adolescent health and development (1–3). This includes substance use, physical violence and self-harm, and risky sexual behaviors (1, 2). The global health burden of such behaviors is substantial. For example, in 2019, self-harm was among the top three causes of death among older adolescent (age 15–19 years) boys and girls, with interpersonal violence also a key cause among boys (6).

Sub-Saharan Africa (SSA) has the highest proportion of adolescents globally (approximately 249 million out of 1.2 billion globally in 2019), a population that is rapidly growing (7). There is a need for more data from this region on the burden of and influences on risky behaviors among adolescents. This includes the potential contribution of depressive disorders and school-going status to the likelihood of engaging in such behaviors. Depressive symptomatology has previously been correlated with increased risk of engaging in risky behaviors among adolescents – but evidence from SSA remains scarce, despite the notable burden of depressive and anxiety disorders documented among adolescents in SSA (1, 8, 9). Additionally, although schools are recognized as important settings for promotion of adolescent health and wellbeing (5), there has been comparatively less research on school-going status as a potentially important factor influencing adolescents’ engagement in risky behaviors particularly in SSA (10, 11). Greater evidence from SSA on the influence of depressive disorders and of school-going status on risky behaviors would be key to support appropriate adolescent health strategies in the region. We sought to examine the potential effect of these two factors on risky behaviors using data from a cross-sectional study based among adolescents in six SSA countries.

## Methods

### Study population

This analysis was based on data from the ARISE Network Adolescent Health Study, which was a cross-sectional community-based survey of adolescents aged 10–19 in nine communities across seven countries: Dar es Salaam, Tanzania (urban); Dodoma, Tanzania (rural); Ningo Prampram, Ghana (rural); Harar, Ethiopia (urban); Kersa, Ethiopia (rural); Ibadan, Nigeria (urban); Nouna, Burkina Faso (rural); Iganga and Mayuge, Uganda (rural), and Lubombo/Manzini, eSwatini (rural). We used data from all sites apart from Lubombo/Manzini, as the study tools used to measure key characteristics including depressive symptoms were different at this site.

The methods for this survey have been described elsewhere (12); a brief summary is as follows. In each community, we used household-based sampling to recruit adolescents. Informed consent (age 18–19 years) or informed assent and parental consent (age 10–17 years) was first obtained. Participants then underwent face-to-face interviews with trained data collectors in private areas within their household or compound. Interviews were conducted in the local language, and used a questionnaire that was adapted from the Global School-Based Health Survey (GSHS) and covered a range of domains such as socio-demographic characteristics, psychosocial health, and nutrition. Ethical approval for the survey was obtained from institutional review boards or human research ethics committees from multiple institutions: the Nouna Health Research Center (Burkina Faso), the University of Heidelberg (Germany), Haramaya University (Ethiopia), the University of Ghana (Ghana), the University of Ibadan (Nigeria), the University of Swaziland (Eswatini), Muhimbili University of Health and Allied Sciences (Tanzania), University of Dodoma (Tanzania), Makerere University (Uganda), and the Harvard T.H. Chan School of Public Health (United States) (12).

### Measures of interest and variable transformation

Outcomes of interest included recent engagement in the following risky behaviors: alcohol use, cigarette or other tobacco product use, being involved in a physical fight, no use of condoms, and suicidal behavior. While some previous literature has made a distinction between risky behaviors and self-harm or suicidal behavior (2), other research has considered self-harm and suicidal behavior to fall under risky behaviors (1). In the current analysis we considered suicidal behavior as a type of risky behavior, given the fact that suicidal behavior is voluntary behavior exposing individuals to substantially increased risk of adverse health consequences or mortality (1). Recent alcohol and cigarette or other tobacco product use were defined as use of these substances in the past 30 days. Recently being involved in a physical fight was defined as getting into a physical fight in the past 12 months. Recent suicidal behavior was defined as making plans to attempt suicide or attempting suicide in the past 12 months. Recently not using condoms was defined as not using condoms the last time the participant had sex (analyses were restricted to participants who reported ever having sex). We also considered ever using drugs, defined as ever using any substance apart from alcohol and tobacco, and including marijuana, khat, cocaine, inhalants or other – although this variable did not strictly measure recent use.

Exposures of interest included depressive symptoms, measured using the six-item Kutcher Adolescent Depression Scale (KADS-6), which probes the frequency of depressive symptoms over the last week (13). The relation between depressive symptoms and risky behaviors has been recognized to potentially be bidirectional (14); however, in the current analysis we aimed to specifically estimate the effect of depressive symptoms on risky behaviors in an effort to explore whether targeting depressive symptoms may result in reducing such behaviors. We examined site-specific tertiles for KADS-6 score, as has been done previously (15), since the KADS-6 has not been validated in the study sites. School-going status was also an exposure of interest, defined as whether or not the participant was currently in school. We also considered potential sociodemographic confounders: sex, age (categorized as 10–14, 15–17 or 18–19 years), site-specific household wealth index tertile (based on a site-specific household wealth score calculated using principal components analysis of key household assets), having both parents alive, living with both parents, and engaging in money-earning activities in the past 12 months. The prevalence of variables of interest (exposures, outcomes, and potential confounders) have been previously examined overall and stratified by sex at the country levels (15, 16); hence we did not focus on country-specific prevalence estimates in this current analysis.

### Statistical analysis

We first examined the distribution of the study population across key characteristics, overall and stratified by sex. Then, we examined the distribution of participant characteristics by recent engagement in each risky behavior. Differences in proportions across groups were assessed using Pearson’s Chi squared tests or Fisher’s Exact tests (comparisons with cell values <5).

We then used mixed-effects Poisson regression models with robust standard errors to examine associations between KADS-6 tertile or school-going status (exposures) and each risky behavior of interest (outcomes). Following preliminary univariable assessments of each of these factors, we constructed fully adjusted models. Fully-adjusted models included both school-going status and depressive symptom tertile, along with all potential confounders noted above, and were additionally adjusted for clustering at the site level. We used the missing indicator method to handle missing data in regression models. As likelihood ratio tests suggested the effect across tertiles of KADS-6 score was not linear for most outcomes, we did not calculate and report *p* values for linear trend for this variable. For school-going status, we examined both models using in-school and those using out-of-school as the referent category, in order to facilitate ease of interpretation.

Finally, we additionally explored effect modification of associations by age, sex and school-going status (the latter variable for KADS-6 tertile only). We did this by (i) using likelihood ratio tests to compare fully-adjusted models with fully-adjusted models additionally including the appropriate interaction term and (ii) examining associations in stratified models (these analyses were restricted to participants with non-missing age, sex and school-going status). All analyses were performed using Stata 16 (StataCorp, Texas).

## Results

Overall, analyses were based among 7,661 adolescents (50.1% girls) (Table 1). About half were 10–14 years of age, 86.4% had both parents alive and 64.8% lived with both parents. About one third of participants engaged in money-earning activities in the past year, and about 70% were in school. Approximately half of participants were in the lowest tertile of site-specific KADS-6 score, with one quarter each in the higher tertiles. Approximately 3% of adolescents recently used cigarettes or tobacco and 4% recently used alcohol; about 5% reported ever using other substances. Just over one quarter had recently engaged in a physical fight. About 8% of adolescents (or 59.8% of those who had ever had sex) reported not recently using a condom. Approximately 2% reported recent suicidal behavior (Table 1).

**Table 1:** Distribution of sociodemographic characteristics, 6-item Kutcher Adolescent Depression Scale (KADS-6) score tertile, and risky behaviors among adolescents.

	*n* (%)			*P*
	Overall	Boys	Girls	
Overall	7,661 (100.0)	3,825 (49.9)	3,836 (50.1)	
Age (years)				
10–14	4,012 (52.4)	1970 (51.5)	2042 (53.2)	
15–17	2,425 (31.7)	1,197 (31.3)	1,228 (32.0)	
18–19	1,224 (16.0)	658 (17.2)	566 (14.8)	0.014
Both parents alive				
No	1,015 (13.2)	524 (13.7)	491 (12.8)	
Yes	6,622 (86.4)	3,285 (85.9)	3,337 (87.0)	0.231
Missing	24 (0.3)	16 (0.4)	8 (0.2)	
Live with both parents				
No	2,668 (34.8)	1,225 (32.0)	1,443 (37.6)	
Yes	4,964 (64.8)	2,594 (67.8)	2,370 (61.8)	<0.001
Missing	29 (0.4)	6 (0.2)	23 (0.6)	
Wealth tertile				
Lowest	2,739 (35.8)	1,363 (35.6)	1,376 (35.9)	
Middle	2,425 (31.7)	1,220 (31.9)	1,205 (31.4)	
Highest	2,477 (32.3)	1,231 (32.2)	1,246 (32.5)	0.894
Missing	20 (0.3)	11 (0.3)	9 (0.2)	
Money earning activity past year				
No	5,069 (66.2)	2,272 (59.4)	2,797 (72.9)	
Yes	2,558 (33.4)	1,541 (40.3)	1,017 (26.5)	<0.001
Missing	34 (0.4)	12 (0.3)	22 (0.6)	
In school				
No	2,228 (29.1)	1,223 (32.0)	1,005 (26.2)	
Yes	5,408 (70.6)	2,591 (67.7)	2,817 (73.4)	<0.001
Missing	25 (0.3)	11 (0.3)	14 (0.4)	
KADS-6 score tertile				
Lowest	3,567 (46.6)	1930 (50.5)	1,637 (42.7)	
Middle	1954 (25.5)	934 (24.4)	1,020 (26.6)	
Highest	1991 (26.0)	890 (23.3)	1,101 (28.7)	<0.001
Missing	149 (1.9)	71 (1.9)	78 (2.0)	
Cigarette or tobacco use				
No	7,247 (94.6)	3,598 (94.1)	3,649 (95.1)	
Yes	223 (2.9)	133 (3.5)	90 (2.3)	0.003
Missing	191 (2.5)	94 (2.5)	97 (2.5)	
Alcohol use				
No	7,315 (95.5)	3,624 (94.7)	3,691 (96.2)	
Yes	317 (4.1)	189 (4.9)	128 (3.3)	<0.001
Missing	29 (0.4)	12 (0.3)	17 (0.4)	
Physical fight				
No	5,567 (72.7)	2,566 (67.1)	3,001 (78.2)	
Yes	2005 (26.2)	1,218 (31.8)	787 (20.5)	<0.001
Missing	89 (1.2)	41 (1.1)	48 (1.3)	
Ever used other substances				
No	2,999 (39.1)	1,415 (37.0)	1,584 (41.3)	
Yes	394 (5.1)	285 (7.5)	109 (2.8)	<0.001
Missing	4,268 (55.7)	2,125 (55.6)	2,143 (55.9)	
No condom use last sexual intercourse				
No	429 (5.6)	226 (5.9)	203 (5.3)	
Yes	640 (8.4)	349 (9.1)	291 (7.6)	0.552
Missing	6,592 (86.0)	3,250 (85.0)	3,342 (87.1)	
Suicidal behavior				
No	5,923 (77.3)	2,873 (75.1)	3,050 (79.5)	
Yes	169 (2.2)	46 (1.2)	123 (3.2)	<0.001
Missing	1,569 (20.5)	906 (23.7)	663 (17.3)	
Site				
Dar es Salaam, Tanzania	824 (10.8)	375 (9.8)	449 (11.7)	
Dodoma, Tanzania	1,226 (16.0)	548 (14.3)	678 (17.7)	
Ningo Prampram, Ghana	625 (8.2)	281 (7.3)	344 (9.0)	
Harar, Ethiopia	1,059 (13.8)	500 (13.1)	559 (14.6)	
Kersa, Ethiopia	951 (12.4)	528 (13.8)	423 (11.0)	
Ibadan, Nigeria	750 (9.8)	339 (8.9)	411 (10.7)	
Nouna, Burkina Faso	1,629 (21.3)	942 (24.6)	687 (17.9)	
Iganga and Mayuge, Uganda	597 (7.8)	312 (8.2)	285 (7.4)	<0.001

*P* for differences across distributions calculated using Pearson’s Chi squared test.

We observed notable sex-specific differences in characteristics, with a greater proportion of adolescent girls aged 10–14 years (*p* = 0.014), in-school (*p* < 0.001) and in the highest tertile of KADS-6 score (*p* < 0.001). Additionally, a smaller proportion of girls compared with boys were living with both parents, engaged in money-earning activities in the past year (*p* < 0.001 for both), and engaged in all risky behaviors (*p* < 0.001 for all except no condom use) (Table 1).

We then examined the distribution of risky behaviors by KADS-6 score tertile and school-going status (Table 2). A notably greater proportion of adolescents who were in the highest KADS-6 score tertile used cigarettes or tobacco, alcohol, or other substances (*p* < 0.001 for all except cigarette or tobacco use). Similarly, a greater proportion of those who were in the highest KADS-6 score tertile recently engaged in a physical fight or in suicidal behavior (*p* < 0.001 for both). Conversely, a smaller proportion of those who were in school used cigarettes or tobacco, alcohol, or other substances (*p* < 0.001 for all), with similar trends also for no condom use (*p* = 0.008) and suicidal behavior (*p* = 0.181). A slightly higher proportion of adolescents who were in school reported getting into a fight (*p* = 0.014) (Table 2).

**Table 2:** Distribution of risky behaviors among adolescents by 6-item Kutcher Adolescent Depression Scale score tertile and school-going status.

	Cigarette or tobacco use (*N* = 7,470)		Alcohol use (*N* = 7,632)		Physical fight (*N* = 7,572)		Ever used other substances (*N* = 3,393)		No condom use last sexual intercourse (*N* = 1,069)		Suicidal behavior (*N* = 6,092)	
	No	Yes	No	Yes	No	Yes	No	Yes	No	Yes	No	Yes
KADS-6 score tertile												
Lowest	3,395 (97.4)	89 (2.6)	3,422 (96.3)	133 (3.7)	2,718 (77.1)	809 (22.9)	1,179 (90.1)	130 (9.9)	150 (37.8)	247 (62.2)	2,419 (99.1)	21 (0.9)
Middle	1847 (96.9)	60 (3.1)	1878 (96.5)	68 (3.5)	1,368 (70.8)	564 (29.2)	951 (90.5)	100 (9.5)	103 (41.0)	148 (59.0)	1905 (98.1)	36 (1.9)
Highest	1887 (96.4)	70 (3.6)	1874 (94.3)	113 (5.7)	1,367 (69.2)	609 (30.8)	759 (82.5)	161 (17.5)	155 (39.7)	235 (60.3)	1,469 (93.1)	109 (6.9)
Missing	118 (96.7)	4 (3.3)	141 (97.9)	3 (2.1)	114 (83.2)	23 (16.8)	110 (97.3)	3 (2.7)	21 (67.7)	10 (32.3)	130 (97.7)	3 (2.3)
*P*	0.092		<0.001		<0.001		<0.001		0.694		<0.001	
In school												
No	2086 (95.9)	89 (4.1)	2075 (93.4)	147 (6.6)	1,654 (75.5)	538 (24.5)	599 (77.0)	179 (23.0)	235 (36.7)	405 (63.3)	1,412 (96.7)	48 (3.3)
Yes	5,142 (97.5)	131 (2.5)	5,216 (96.8)	170 (3.2)	3,895 (72.7)	1,462 (27.3)	2,386 (91.8)	213 (8.2)	190 (44.8)	234 (55.2)	4,486 (97.4)	121 (2.6)
Missing	19 (86.4)	3 (13.6)	24 (100.0)	0 (0.0)	18 (78.3)	5 (21.7)	14 (87.5)	2 (12.5)	4 (80.0)	1 (20.0)	25 (100.0)	0 (0.0)
*P*	<0.001		<0.001		0.014		<0.001		0.008		0.181	

*P* for differences across distributions (excluding missing categories) calculated using Pearson’s Chi squared test.

Following this, we assessed the potential effect of KADS-6 score tertile on risky behaviors in multivariable mixed-effects Poisson regression models (Table 3). Broadly, we observed successive increases in the risk of engaging in almost all risky behaviors associated with increases in KADS-6 score tertile – with the exception of no condom use. Compared with the lowest KADS-6 score tertile, being in the highest tertile was associated with 25% increased risk of cigarette and tobacco use [adjusted risk ratio (RR): 1.25, 95% confidence interval (95% CI): 1.06–1.48, *p* = 0.009], 70% higher risk of alcohol use (RR: 1.70, 95% CI: 1.48–1.95, *p* < 0.001), and 42% higher risk of using other substances (RR: 1.42, 95% CI: 1.02–1.98, *p* = 0.039). Progressive increases in risk were also observed for engaging in a physical fight (RR for highest KADS-6 score tertile: 1.52, 95% CI: 1.36–1.70, *p* < 0.001). The risk of engaging in suicidal behavior was almost double among adolescents in the middle versus the lowest KADS-6 tertile (RR: 1.92, 95% CI: 1.01–3.65, *p* = 0.046), and seven times as high in the highest tertile (RR: 7.07, 95% CI: 2.69–18.57, *p* < 0.001) (Table 3).

**Table 3:** Association between 6-item Kutcher Adolescent Depression Scale score (KADS-6) tertile and risky behaviors among adolescents.

	Adjusted risk ratio (95% confidence interval)	*P*
Cigarette or tobacco use (*N* = 7,470)		
Lowest	1.00	
Middle	1.03 (0.73–1.44)	0.882
Highest	1.25 (1.06–1.48)	0.009
Alcohol use (*N* = 7,632)		
Lowest	1.00	
Middle	1.54 (1.20–1.97)	<0.001
Highest	1.70 (1.48–1.95)	<0.001
Physical fight (*N* = 7,572)		
Lowest	1.00	
Middle	1.35 (1.14–1.59)	<0.001
Highest	1.52 (1.36–1.70)	<0.001
Ever used other substances (*N* = 3,393)		
Lowest	1.00	
Middle	1.04 (0.90–1.20)	0.585
Highest	1.42 (1.02–1.98)	0.039
No condom use last sexual intercourse (*N* = 1,069)		
Lowest	1.00	
Middle	0.99 (0.88–1.11)	0.834
Highest	1.03 (0.88–1.21)	0.683
Suicidal behavior (*N* = 6,092)		
Lowest	1.00	
Middle	1.92 (1.01–3.65)	0.046
Highest	7.07 (2.69–18.57)	<0.001

Estimates based on mixed-effects Poisson regression models adjusted for age, sex, wealth tertile, both parents alive, living with both parents, undertaking money-earning activities within the past 12 months, and school-going status, and for clustering at site level. *P* (trend) not calculated as likelihood ratio tests suggested statistical difference (*p* < 0.05) between KADS-6 tertile as a continuous or categorical variable for many outcomes.

We similarly examined the relation between school-going status and risky behaviors in multivariable models (Table 4; Supplementary Table S2). Currently being in school was associated with approximately 25%–39% reduced risk of engaging in substance use, although associations were not as statistically strong as those observed for depressive symptoms (RR for cigarette or tobacco use: 0.61, 95% CI: 0.40–0.93, *p* = 0.021, for alcohol use: 0.73, 95% CI: 0.53–1.00, *p* = 0.047; for other substance use: 0.75, 95% CI: 0.59–0.95, *p* = 0.016). In addition, being in school was associated with lower risk of not using a condom (RR: 0.81, 95% CI: 0.66–0.99, *p* = 0.040). No clear associations were observed for engaging in a physical fight and for suicidal behavior (Table 4). Corresponding associations between being out of school and risky behaviors (i.e., using in-school as the referent category) are summarized in Supplementary Table S2.

**Table 4:** Association between school-going status and risky behaviors among adolescents.

	Adjusted risk ratio (95% confidence interval)	*P*
Cigarette or tobacco use (*N* = 7,470)		
Out of school	1.00	
In school	0.61 (0.40–0.93)	0.021
Alcohol use (*N* = 7,632)		
Out of school	1.00	
In school	0.73 (0.53–1.00)	0.047
Physical fight (*N* = 7,572)		
Out of school	1.00	
In school	1.03 (0.94–1.13)	0.506
Ever used other substances (*N* = 3,393)		
Out of school	1.00	
In school	0.75 (0.59–0.95)	0.016
No condom use last sexual intercourse (*N* = 1,069)		
Out of school	1.00	
In school	0.81 (0.66–0.99)	0.040
Suicidal behavior (*N* = 6,092)		
Out of school	1.00	
In school	0.95 (0.71–1.26)	0.707

Estimates based on mixed-effects Poisson regression models adjusted for age, sex, wealth tertile, both parents alive, living with both parents, undertaking money-earning activities within the past 12 months, and school-going status, and for clustering at site level.

Finally, we examined potential effect modification of associations by age, sex and school-going status (Table 5 and Supplementary Figures S1–S3). There was evidence of modification of associations between school-going status and certain risky behaviors by sex. This included cigarette or tobacco use (*P* for interaction <0.001) and suicidal behavior (*P* for interaction = 0.013), where the protective effect of being in school was observed only for boys (Table 5 and Supplementary Figure S1). Additionally, in stratified models, being in school was associated with increased risk of getting into a physical fight (*P* for interaction: 0.003) and decreased risk of not using a condom (*P* for interaction: 0.014) among girls only, with no or opposite associations for boys. Statistical evidence of interaction between sex and KADS-6 score tertile was less clear, although the risk of suicidal behavior associated with higher KADS-6 score tertiles appeared to be noticeably greater among girls (*P* for interaction: 0.041) (Table 5 and Supplementary Figure S1).

**Table 5:** *P* values for interaction between 6-item Kutcher Adolescent Depression Scale (KADS-6) score tertile or school-going status and sociodemographic characteristics (sex, age, and school-going status).

Outcome	Predictor	*P* value for interaction with variable		
		Sex	Age	In school
Cigarette or tobacco use	KADS-6 score tertile	0.073	0.532	0.046
	School-going status	<0.001	0.002	–
Alcohol use	KADS-6 score tertile	0.063	0.006	0.404
	School-going status	0.197	0.125	–
Physical fight	KADS-6 score tertile	0.568	0.161	0.751
	School-going status	0.003	0.037	–
Ever used other substances	KADS-6 score tertile	0.097	0.222	0.850
	School-going status	0.402	0.002	–
No condom use last sexual intercourse	KADS-6 score tertile	0.854	0.570	0.645
	School-going status	0.014	0.010	–
Suicidal behavior	KADS-6 score tertile	0.041	0.318	0.133
	School-going status	0.013	0.317	–

Estimates based on fixed-effects Poisson regression models adjusted for age, sex, wealth quintile, mother’s and father’s education, undertaking money-earning activities within the past 12 months. *P* values of likelihood ratio tests comparing these fully adjusted models with models additionally including an interaction term between KADS-6 score tertile or school-going status and potential effect modifier of interest.

Data also suggested modification of the effect of school-going status by age (Table 5 and Supplementary Figure S2). In models stratified by age, a protective effect of being in school on both cigarette or tobacco use and other substance use was observed only for adolescents aged 15–17 years (*P* for interaction: 0.002 for both). Decreased risk of no condom use associated with being in school was observed only for adolescents aged 18–19 years (*P* for interaction: 0.010). On the other hand, being in school was associated with a marginally higher risk of getting into a physical fight among adolescents aged 10–14, but not among other age groups (*P* for interaction: 0.037). Evidence of interaction between age and KADS-6 score was again less clear apart from for alcohol use, where positive associations were observed only for the younger adolescent age groups (10–14 and 15–17) with higher KADS-score (*P* for interaction: 0.006) (Table 5 and Supplementary Figure S2).

There was some indication that the risk of suicidal behavior among adolescents in higher tertiles of KADS-6 score may be greater among those who were in-school; however, this was not statistically notable (*P* for interaction: 0.317) (Table 5 and Supplementary Figure S3). We found no clear evidence of interaction between KADS-6 score tertile and school-going status for any other outcome (Table 5 and Supplementary Figure S3).

## Discussion

There is a need for greater evidence from SSA populations regarding risky behaviors during adolescence. In this analysis, we assessed potential influences of depressive symptoms and school-going status on risky behaviors among adolescents aged 10–19 years in six SSA countries. The overall burden of risky behaviors in this population varied, with a small but notable proportion engaging in substance use, one quarter engaging in a physical fight, over half of those who had ever had sex reporting no condom use on the most recent occasion, and 2% reporting recent suicidal behavior. Depressive symptoms were independently associated with increased risk of all risky behaviors, with the exception of no condom use. On the other hand, being in school was associated with decreased risk of using substances and decreased risk of no condom use. We observed evidence of modification of the effect of school-going status on multiple risky behaviors by age and sex. These data reinforce the need for continued focus on risky behaviors among adolescents in SSA, and point towards potentially important approaches that may help inform relevant interventions.

Previous studies have indicated a notable burden of risky behaviors across SSA. Evidence has commonly been based on small sample sizes (17–19), and often focused on specific sub-populations, such as school-going adolescents (17), or adolescents living with HIV (20). Among identified studies examining general (often in-school) adolescent populations, recent estimates suggest prevalence as high as over 25% for cigarette use, over 40% for alcohol use, and over 50% for use of other substances (18, 19, 21). The estimated prevalence of suicidal behavior among adolescents ranged from around 12–15% in a Ugandan population to over 25% in a Ghanaian population (22–24). Risky sexual behaviors including no or inconsistent condom use have been commonly reported, with recent estimates from studies including DHS surveys suggesting 50–60% prevalence across multiple SSA adolescent populations (25, 26). Although studies on violence against adolescents were numerous, we found comparatively less evidence examining physical violence perpetrated by adolescents, with one study based in South Africa suggesting a prevalence of getting into fights of about 5% and physically attacking others of about 2% (27). The crude prevalence of risky behaviors in the current analysis adds to these estimates, suggesting a notable recent burden of risky behaviors in both in-school and out of school adolescents across multiple countries, and indicating the need to better address these.

There is some existing evidence from SSA suggesting associations between depressive symptoms or other measures of poor psychosocial status and increased likelihood of engaging in risky behaviors. Much of these data are from in-school cross-sectional surveys based among adolescents, and include studies indicating positive associations between symptoms of depression or anxiety and suicidal behaviors (1, 22–24). Studies have also suggested the influence of adolescent depressive symptoms or psychosocial distress on risk of heavy alcohol use, other drug use, or having substance use disorders (1, 18, 21). Similarly, some previous studies have examined the influence of depressive symptoms on engagement in risky sexual behaviors, with those identified here suggesting such symptoms were associated with increased risk of not using condoms among adolescents and young people (17, 28), and increased risk of adolescent pregnancy (1). Adolescents with depressive symptoms are thought to be more likely to engage in risky behaviors as a way of coping with low mood (1, 29), and also as a result of changes in relevant cognitive functions, such as inhibitory control, that are associated with depression (1). Our findings provide further evidence to support previously reported patterns, replicating strong associations between depressive symptoms and a range of risky behaviors in a large, multi-country population. Consistent with these reports, we found positive associations between depressive symptoms and all risky behaviors examined here except no condom use. Collectively, these data suggest that targeting depressive symptoms may be an important way of reducing adolescent engagement in risky behaviors. Importantly, this would result in dual gains for adolescent health – in terms of both improved mental health but also reduced risk of health consequences associated with risky behaviors.

Little research has previously examined the association of school-going status on risky behaviors in SSA adolescent populations. Of the limited available evidence identified here, all was based on cross-sectional data. One study based in a Tanzanian population aged 10–19 years indicated that in-school adolescents were more likely to exhibit risky sexual behaviors (25), while another study suggested that being out of school was associated with increased risk of alcohol and cigarette use among adolescents in Johannesburg (19). We identified no relevant data on the association between school-going status and other outcomes including suicidal behavior and engaging in a physical fight. In the current analysis, we observed reduced likelihood of key risky behaviors among in-school adolescents compared with out-of-school adolescents, including substance use and not using a condom. Schools have long been recognized as important platforms for health promotion and delivery of health-related interventions among children and adolescents (5). While in this survey we did not collect information on the existence of health-specific programmes in school, the observed protective effects of substance use and condom use may be the result of both direct influences such as school-based sexual and reproductive health education, and possibly other indirect effects of the general school environment (30). Importantly, this analysis provides further evidence to support the potential of improving school attendance, and building on schools as platforms for the delivery of simple interventions, to improve adolescent health.

To our knowledge, there is little to no previous SSA-based evidence on the potential modifying effect of key demographic characteristics on the influence of depressive symptoms and school-going status on adolescent risky behaviors. As part of a literature search for the current analysis, we identified no studies comprehensively examining the same. Evidence from this analysis indicates key interactions between adolescents’ school-going status and sex or age on risky behaviors. For example, protective effects of school-going on cigarette or tobacco use and on suicidal behavior were only observed among boys, while protective effects on no condom use were observed among girls and among older adolescents aged 18–19 years. Conversely, being in school was associated with increased risk of engaging in a physical fight among girls, and among younger adolescents aged 10–14 years. These data highlight the need for future studies to more closely examine potential sub-group specific differences regarding influences on adolescent risky behaviors, and related mechanisms. They preliminarily indicate that school-based approaches to target risky behaviors may be more effective if closely tailored to specific subgroups.

The current analysis has multiple strengths. It is based on a large and detailed community-based survey covering both in-school and out-of-school adolescents aged 10–19 across eight communities in six SSA countries. The available data and large overall sample size enabled the examination of a wide range of risky behaviors, and also the assessment of differences in associations across key demographic subgroups. Previous analyses based on these data have summarized country-specific estimates of prevalence for all measures examined here, outlining a substantial proportion of adolescents being out of school and a notable burden of depressive symptoms and risky behaviors, across generally all countries (15, 16). While those estimates are key to guiding national priorities for adolescent health, the current analyses indicate potentially important population-level associations to consider when designing appropriate strategies to address these health-related issues. Additionally, the data summarized here provide an important reference of patterns in adolescent health risks prior to the COVID-19 pandemic, valuable to understanding how these health risks have changed in SSA since this key disruption. An important limitation is the cross-sectional design, which limits inferences about causality as we are unable to establish the temporal nature of associations. Importantly, associations between mental health and certain risky behaviors may be bidirectional (14) – which we were unable to parse given the cross-sectional design. Further, longitudinal analyses examining this potential bidirectionality in greater detail would be key to better informing the design of strategies to improve mental health and risky behaviors in these populations. Additionally, we did not collect information on specific money-earning activities, which could be potentially key to explaining risky behaviors such as unprotected sex among girls in settings where transactional sex may be more common (31). Additionally, sample sizes for certain outcomes such as no condom use during last sexual intercourse were reduced, limiting statistical power. Due to these reduced sample sizes, we were unable to examine other potentially important aspects, such as the possible clustering of multiple risky behaviors (10, 22). Nonetheless, the current analysis provides an important evidence base to pursue further longitudinal and interventional SSA-based research comprehensively investigating the influence of depressive symptoms and school-going status on adolescent risky behaviors.

To conclude, in this multi-country SSA-based study, we observed independent influences of depressive symptoms and school-going status on multiple adolescent risky behaviors – with depressive symptoms generally associated with increased risk of these behaviors, and school-going status associated with protective effects. Our findings reinforce the need for a greater public health focus on risky behaviors among adolescents in SSA. Addressing depressive symptoms among adolescents, and facilitating school attendance and using schools as platforms to improve health may help reduce risky behaviors in this and similar populations.

## Data availability statement

The original contributions presented in the study are included in the article/Supplementary material, further inquiries can be directed to the corresponding author.

## Ethics statement

The studies involving human participants were reviewed and approved by the Nouna Health Research Center (Burkina Faso), the University of Heidelberg (Germany), Haramaya University (Ethiopia), the University of Ghana (Ghana), the University of Ibadan (Nigeria), the University of Swaziland (Eswatini), Muhimbili University of Health and Allied Sciences (Tanzania), University of Dodoma (Tanzania), Makerere University (Uganda) and the Harvard T.H. Chan School of Public Health (United States). Written informed consent to participate in this study was provided by the participants’ legal guardian/next of kin.

## Author contributions

WF, TB, NA, YB, AS, DG, JK, AO, MS, SV, and RA were involved in the original study design and data collection. UP conceptualized the analysis, performed statistical analyses, and wrote the first draft of the manuscript with critical input from WF. All authors contributed to the article and approved the submitted version.

## Funding

Funding for the ARISE Adolescent Health Study was provided by the Department of Global Health and Population at Harvard T.H. Chan School of Public Health.

## Conflict of interest

The authors declare that the research was conducted in the absence of any commercial or financial relationships that could be construed as a potential conflict of interest.

## Publisher’s note

All claims expressed in this article are solely those of the authors and do not necessarily represent those of their affiliated organizations, or those of the publisher, the editors and the reviewers. Any product that may be evaluated in this article, or claim that may be made by its manufacturer, is not guaranteed or endorsed by the publisher.
