# Supplementary material for: The influence of depressive symptoms and school-going status on risky behaviors: a pooled analysis among adolescents in six sub-Saharan African countries
Source: Front Psychiatry. 2023 Jul 24;14:1171231. doi: 10.3389/fpsyt.2023.1171231 (PMC10406520; doi:10.3389/fpsyt.2023.1171231)
Supplement: Supplementary file 1 [file Data_Sheet_1.docx]

**Supplementary Tables and Figures**

| **Supplementary Table 1. Distribution of sociodemographic characteristics and 6-item Kutcher Adolescent Depression Scale (KADS-6) score tertile by risky behaviors among adolescents.** | | | | | | | | | | | | |
| --- | --- | --- | --- | --- | --- | --- | --- | --- | --- | --- | --- | --- |
|  | **Cigarette or tobacco use (N=7470)** | | **Alcohol use (N=7632)** | | **Physical fight (N=7572)** | | **Ever used other substances (N=3393)** | | **No condom use last sexual intercourse (N=1069)** | | **Suicidal behavior (N=6092)** | |
|  | **No** | **Yes** | **No** | **Yes** | **No** | **Yes** | **No** | **Yes** | **No** | **Yes** | **No** | **Yes** |
|  |  |  |  |  |  |  |  |  |  |  |  |  |
| **Sex** | | |  |  |  |  |  |  |  |  |  |  |
| **Boys** | 3598 (49.6) | 133 (59.6) | 3624 (49.5) | 189 (59.6) | 2566 (46.1) | 1218 (60.7) | 1415 (47.2) | 285 (72.3) | 226 (52.7) | 349 (54.5) | 2873 (48.5) | 46 (27.2) |
| **Girls** | 3649 (50.4) | 90 (40.4) | 3691 (50.5) | 128 (40.4) | 3001 (53.9) | 787 (39.3) | 1584 (52.8) | 109 (27.7) | 203 (47.3) | 291 (45.5) | 3050 (51.5) | 123 (72.8) |
| ***P*** | 0.003 |  | <0.001 |  | <0.001 |  | <0.001 |  | 0.552 |  | <0.001 |  |
| **Age (years)** | |  |  |  |  |  |  |  |  |  |  |  |
| **10-14** | 3858 (53.2) | 87 (39.0) | 3903 (53.4) | 96 (30.3) | 2693 (48.4) | 1289 (64.3) | 1706 (56.9) | 94 (23.9) | 19 (4.4) | 136 (21.3) | 3233 (54.6) | 63 (37.3) |
| **15-17** | 2286 (31.5) | 66 (29.6) | 2302 (31.5) | 114 (36.0) | 1882 (33.8) | 510 (25.4) | 868 (28.9) | 167 (42.4) | 147 (34.3) | 231 (36.1) | 1819 (30.7) | 64 (37.9) |
| **18-19** | 1103 (15.2) | 70 (31.4) | 1110 (15.2) | 107 (33.8) | 992 (17.8) | 206 (10.3) | 425 (14.2) | 133 (33.8) | 263 (61.3) | 273 (42.7) | 871 (14.7) | 42 (24.9) |
| ***P*** | <0.001 |  | <0.001 |  | <0.001 |  | <0.001 |  | <0.001 |  | <0.001 |  |
| **Both parents alive** | |  |  |  |  |  |  |  |  |  |  |  |
| **No** | 949 (13.1) | 36 (16.1) | 966 (13.2) | 46 (14.5) | 744 (13.4) | 258 (12.9) | 425 (14.2) | 82 (20.8) | 94 (21.9) | 112 (17.5) | 803 (13.6) | 41 (24.3) |
| **Yes** | 6274 (86.6) | 187 (83.9) | 6325 (86.5) | 271 (85.5) | 4800 (86.2) | 1746 (87.1) | 2559 (85.3) | 310 (78.7) | 335 (78.1) | 527 (82.3) | 5096 (86.0) | 128 (75.7) |
| **Missing** | 24 (0.3) | 0 (0.0) | 24 (0.3) | 0 (0.0) | 23 (0.4) | 1 (0.0) | 15 (0.5) | 2 (0.5) | (0.0) | 1 (0.2) | 24 (0.4) | 0 (0.0) |
| ***P*** | 0.192 |  | 0.517 |  | 0.537 |  | <0.001 |  | 0.075 |  | <0.001 |  |
| **Live with both parents** | |  |  |  |  |  |  |  |  |  |  |  |
| **No** | 2518 (34.7) | 77 (34.5) | 2526 (34.5) | 133 (42.0) | 1889 (33.9) | 743 (37.1) | 908 (30.3) | 124 (31.5) | 214 (49.9) | 336 (52.5) | 2117 (35.7) | 84 (49.7) |
| **Yes** | 4703 (64.9) | 146 (65.5) | 4762 (65.1) | 182 (57.4) | 3655 (65.7) | 1256 (62.6) | 2084 (69.5) | 270 (68.5) | 214 (49.9) | 292 (45.6) | 3778 (63.8) | 85 (50.3) |
| **Missing** | 26 (0.4) | 0 (0.0) | 27 (0.4) | 2 (0.6) | 23 (0.4) | 6 (0.3) | 7 (0.2) | (0.0) | 1 (0.2) | 12 (1.9) | 28 (0.5) | 0 (0.0) |
| ***P*** | 0.916 |  | 0.006 |  | 0.013 |  | 0.649 |  | 0.263 |  | <0.001 |  |
| **Wealth tertile** | |  |  |  |  |  |  |  |  |  |  |  |
| **Lowest** | 2599 (35.9) | 75 (33.6) | 2608 (35.7) | 130 (41.0) | 1960 (35.2) | 738 (36.8) | 1135 (37.8) | 140 (35.5) | 127 (29.6) | 233 (36.4) | 2150 (36.3) | 73 (43.2) |
| **Middle** | 2307 (31.8) | 62 (27.8) | 2312 (31.6) | 107 (33.8) | 1716 (30.8) | 679 (33.9) | 900 (30.0) | 130 (33.0) | 135 (31.5) | 213 (33.3) | 1856 (31.3) | 43 (25.4) |
| **Highest** | 2323 (32.1) | 85 (38.1) | 2377 (32.5) | 78 (24.6) | 1877 (33.7) | 582 (29.0) | 947 (31.6) | 123 (31.2) | 163 (38.0) | 189 (29.5) | 1898 (32.0) | 52 (30.8) |
| **Missing** | 18 (0.2) | 1 (0.4) | 18 (0.2) | 2 (0.6) | 14 (0.3) | 6 (0.3) | 17 (0.6) | 1 (0.3) | 4 (0.9) | 5 (0.8) | 19 (0.3) | 1 (0.6) |
| ***P*** | 0.144 |  | 0.012 |  | <0.001 |  | 0.466 |  | 0.009 |  | 0.131 |  |
| **Money earning activity past year** | |  |  |  |  |  |  |  |  |  |  |  |
| **No** | 4793 (66.1) | 130 (58.3) | 4906 (67.1) | 138 (43.5) | 3750 (67.4) | 1249 (62.3) | 2496 (83.2) | 262 (66.5) | 200 (46.6) | 284 (44.4) | 4327 (73.1) | 117 (69.2) |
| **Yes** | 2426 (33.5) | 92 (41.3) | 2380 (32.5) | 175 (55.2) | 1796 (32.3) | 744 (37.1) | 486 (16.2) | 130 (33.0) | 227 (52.9) | 353 (55.2) | 1563 (26.4) | 51 (30.2) |
| **Missing** | 28 (0.4) | 1 (0.4) | 29 (0.4) | 4 (1.3) | 21 (0.4) | 12 (0.6) | 17 (0.6) | 2 (0.5) | 2 (0.5) | 3 (0.5) | 33 (0.6) | 1 (0.6) |
| ***P*** | 0.015 |  | <0.001 |  | <0.001 |  | <0.001 |  | 0.469 |  | 0.269 |  |
| **In school** | |  |  |  |  |  |  |  |  |  |  |  |
| **No** | 2086 (28.8) | 89 (39.9) | 2075 (28.4) | 147 (46.4) | 1654 (29.7) | 538 (26.8) | 599 (20.0) | 179 (45.4) | 235 (54.8) | 405 (63.3) | 1412 (23.8) | 48 (28.4) |
| **Yes** | 5142 (71.0) | 131 (58.7) | 5216 (71.3) | 170 (53.6) | 3895 (70.0) | 1462 (72.9) | 2386 (79.6) | 213 (54.1) | 190 (44.3) | 234 (36.6) | 4486 (75.7) | 121 (71.6) |
| **Missing** | 19 (0.3) | 3 (1.3) | 24 (0.3) | 0 (0.0) | 18 (0.3) | 5 (0.2) | 14 (0.5) | 2 (0.5) | 4 (0.9) | 1 (0.2) | 25 (0.4) | 0 (0.0) |
| ***P*** | <0.001 |  | <0.001 |  | 0.014 |  | <0.001 |  | 0.008 |  | 0.181 |  |
| **KADS-6 score tertile** | |  |  |  |  |  |  |  |  |  |  |  |
| **Lowest** | 3395 (46.8) | 89 (39.9) | 3422 (46.8) | 133 (42.0) | 2718 (48.8) | 809 (40.3) | 1179 (39.3) | 130 (33.0) | 150 (35.0) | 247 (38.6) | 2419 (40.8) | 21 (12.4) |
| **Middle** | 1847 (25.5) | 60 (26.9) | 1878 (25.7) | 68 (21.5) | 1368 (24.6) | 564 (28.1) | 951 (31.7) | 100 (25.4) | 103 (24.0) | 148 (23.1) | 1905 (32.2) | 36 (21.3) |
| **Highest** | 1887 (26.0) | 70 (31.4) | 1874 (25.6) | 113 (35.6) | 1367 (24.6) | 609 (30.4) | 759 (25.3) | 161 (40.9) | 155 (36.1) | 235 (36.7) | 1469 (24.8) | 109 (64.5) |
| **Missing** | 118 (1.6) | 4 (1.8) | 141 (1.9) | 3 (0.9) | 114 (2.0) | 23 (1.1) | 110 (3.7) | 3 (0.8) | 21 (4.9) | 10 (1.6) | 130 (2.2) | 3 (1.8) |
| ***P*** | 0.092 |  | <0.001 |  | <0.001 |  | <0.001 |  | 0.694 |  | <0.001 |  |
|  |  |  |  |  |  |  |  |  |  |  |  |  |
| *P* for differences across distributions (excluding missing categories) calculated using Pearson's Chi squared test. | | | | | | | | | | | | |

| **Supplementary Table 2. Association between school-going status and risky behaviors among adolescents (referent group: in-school).** | | |
| --- | --- | --- |
|  | **Adjusted risk ratio (95% confidence interval)** | ***P*** |
|  |  |  |
| **Cigarette or tobacco use (N=7470)** |  |  |
| **Out of school** | 1.63 (1.08-2.48) |  |
| **In school** | 1.00 | 0.021 |
|  |  |  |
| **Alcohol use (N=7632)** |  |  |
| **Out of school** | 1.37 (1.00-1.88) |  |
| **In school** | 1.00 | 0.047 |
|  |  |  |
| **Physical fight (N=7572)** |  |  |
| **Out of school** | 0.97 (0.89-1.06) |  |
| **In school** | 1.00 | 0.506 |
|  |  |  |
| **Ever used other substances (N=3393)** |  |  |
| **Out of school** | 1.34 (1.06-1.70) |  |
| **In school** | 1.00 | 0.016 |
|  |  |  |
| **No condom use last sexual intercourse (N=1069)** |  |  |
| **Out of school** | 0.77 (0.59-0.99) |  |
| **In school** | 1.00 | 0.040 |
|  |  |  |
| **Suicidal behavior (N=6092)** |  |  |
| **Out of school** | 1.06 (0.80-1.42) |  |
| **In school** | 1.00 | 0.707 |
|  |  |  |
| Estimates based on mixed-effects Poisson regression models adjusted for age, sex, wealth tertile, both parents alive, living with both parents, undertaking money-earning activities within the past 12 months, and school-going status, and for clustering at site level. | | |

**Supplementary Figure 1.** Association between 6-item Kutcher Adolescent Depression Scale (KADS-6) score tertile or school-going status and risky behaviors, stratified by sex.

Estimates from mixed-effects Poisson regression models as described in Tables 2 and 3.

**Supplementary Figure 3.** Association between 6-item Kutcher Adolescent Depression Scale (KADS-6) tertile or school-going status and risky behaviors, stratified by sex.

Estimates from mixed-effects Poisson regression models as described in Tables 2 and 3.

**Supplementary Figure 2 (previous page).** Association between 6-item Kutcher Adolescent Depression Scale (KADS-6) score tertile or school-going status and risky behaviors, stratified by age.

Estimates from mixed-effects Poisson regression models as described in Tables 2 and 3.
